# Teenagers’ and parental attitudes towards the use of placebo pills

**DOI:** 10.1007/s00431-022-04801-4

**Published:** 2023-01-06

**Authors:** Clara M.A. de Bruijn, Marc A. Benninga, Arine M. Vlieger

**Affiliations:** 1 Emma Children’s Hospital, Amsterdam UMC, University of Amsterdam, Pediatric Gastroenterology, Hepatology and Nutrition, Room C2-312, PO Box 22700, Amsterdam, 1100 DD The Netherlands; 2 Department of Pediatrics, St. Antonius Hospital, Nieuwegein, The Netherlands

**Keywords:** Pediatric care, Placebo

## Abstract

The placebo response a significant therapeutic improvement after a placebo intervention — can be high in children. The question arises of how optimal advantages of placebo treatment in pediatric clinical care be achieved. In this era of shared-decision making, it is important to be aware of patients’ and parental attitudes. Therefore, the aim of the current study was to assess teenagers’ and parental views on the use of placebo pills in pediatric clinical care. All patients (aged 12–18 years) and parents of children (aged 0–18 years), visiting the pediatric outpatient clinic between March 2020 through December 2020, were invited to participate in this study multicenter survey study. Of 1644 distributed questionnaires: 200/478 (47%) teenagers and 456/1166 (45%) parents filled out the complete survey. More parents were positive towards prescribing placebo medication than teenagers (80% vs. 71%, *p* = .019), especially when the clinician disclosed the use of a placebo to parents and teenagers, respectively (76% vs. 55%, *p* = .019). Increasing age of teenagers was positively associated with the willingness for placebo interventions (OR 0.803, 95%CI 0.659–0.979), as was a higher level of parental education (OR 0.706, 95%CI 0.526–0.949).

*  Conclusion*: This study emphasizes the willingness of teenagers and parents to receive placebo medication. Placebo medication becoming more acceptable and integrated into daily care may contribute to a decrease in medication use.

**Table:** 

**What is Known:** *• A placebo is a treatment without inherent power to produce any therapeutic effect, but can result in significant therapeutic improvement, the so-called placebo response.* *• Treatment response rates after placebo interventions in children can be high, ranging from 41 to 46% in pediatric trials.*	
**What is New:** *• Most teenagers (71%) and parents (80%) find it appropriate for healthcare professionals to prescribe placebo medication.* *• Compared to adult care, pediatrics has a unique feature to disclose placebo treatment to parents while concealing it for the young patient: the majority of teenagers (85%) and parents (91%) agree to disclose placebo treatment to parents exclusively.*

**What is Known:**

*• A placebo is a treatment without inherent power to produce any therapeutic effect, but can result in significant therapeutic improvement, the so-called placebo response.*

*• Treatment response rates after placebo interventions in children can be high, ranging from 41 to 46% in pediatric trials.*

**What is New:**

*• Most teenagers (71%) and parents (80%) find it appropriate for healthcare professionals to prescribe placebo medication.*

*• Compared to adult care, pediatrics has a unique feature to disclose placebo treatment to parents while concealing it for the young patient: the majority of teenagers (85%) and parents (91%) agree to disclose placebo treatment to parents exclusively.*

## Brief report

A placebo is a substance or procedure without inherent power to produce any therapeutic effect [1]. Nevertheless, it can result in significant therapeutic improvement, the so-called *placebo response*. This response includes both an actual *placebo effect* as well as other factors, like spontaneous improvements and regression to the mean [2]. The underlying neurobiological mechanism of the actual *placebo effect* is likely multifactorial, involving a complex interaction between patients’ beliefs and expectations, social and physical environmental perceptions, and conditioning from past experiences [3, 4]. Treatment success rates after placebo interventions in children can be high, with pooled placebo response rates of 41% and 46% in pediatric functional abdominal pain and pediatric migraine trials, respectively [5, 6]. It is therefore not surprising that many physicians would like to prescribe placebo medication in daily practice, especially to patients with chronic pain and functional disorders [7]. The question arises of how optimal advantages of placebo treatment in clinical care be achieved. A physician can openly discuss the use of placebo medication. However, studies have demonstrated varying treatment effects in patients depending on the labeling of the treatment with lower effects when pills were labeled as placebo (so-called open-label placebo) and higher when pills had an uncertain labeling (i.e., active medication or placebo) [8]. On the other hand, withholding information that a placebo is prescribed raises strong ethical concerns. In this era of shared-decision making, it is important to be aware of patients’ and parental attitudes towards open or covered placebo prescriptions. Surprisingly little attention has been paid to this topic in pediatrics. To date, only one study in the USA has investigated parental views on the clinical use of placebo pills for their child [9]. Hence, we wanted to examine the opinion of parents but also of teenagers, since the latter, by law, fully participate in shared decision-making. Therefore, the aim of the current study was to assess teenagers’ and parental views on the use of placebo pills in pediatric clinical care.

The current study was an ancillary study to an original survey on teenagers’ and parental individual needs for side effects information and the influence of nocebo effect education [10]. This multicenter, cross-sectional survey study was conducted in the Netherlands between March 2020 through December 2020 and enrolled 226 teenagers aged 12–18 years and 525 parents of children aged 0–18 years with an appointment at the pediatric outpatient clinic of a secondary and tertiary medical center. Questionnaires were filled out during their stay at the outpatient clinic. Due to the coronavirus pandemic in 2019 (COVID-19), many consultations were done by phone. These patients received the questionnaires with an information letter at home. Teenagers and parents were asked to complete the questionnaire and return it to the hospital. Questionnaires completed by teenagers were not paired to questionnaires completed by their parents.

For the current ancillary – and original survey – study, a questionnaire was developed assessing demographics and clinical characteristics and teenagers’ and parental attitudes on the use of placebo pills in pediatric clinical care and whether the doctor should be open about prescribing placebos. To test for validity, the draft version of the questionnaire was tested in five teenagers and five parents to ensure that teenagers and parents understood the content and interpreted the questions similarly. This resulted in minor adjustments only. The required time to fulfill the complete survey was approximately 15 min. Before attitudes were assessed, participants were briefly informed about the concept of placebo medication (Table 1). The full version of the questionnaire is accessible upon request (only available in Dutch).

**Table 1:** Placebo pill education

**From medical research doctors know that almost half of the patients with, for example, chronic pain or other complaints, will improve after taking fake pills — also called placebo pills. These fake pills have no side effects because they are like “sugar pills.” If patients know in advance that their medication is a placebo, the chances of symptom improvement are less**
**With this knowledge, do you think your pediatrician could prescribe you/your child a placebo pill for 2 weeks to explore if your (child’s) symptoms will improve?**
◦ Yes, the pediatrician could prescribe a placebo pill and not tell both patient and parents that the treatment is a placebo (concealed placebo)
◦ Yes, but only when the pediatrician tells both patient and parents that the treatment is a placebo, even if the chance of symptom improvement becomes less
◦ Yes, but only when the pediatrician tells the parents but not the child that the treatment is a placebo
◦ No, pediatricians should not prescribe a placebo pill, they should always be honest

Descriptive statistics were used to summarize variables. Multivariable logistic analyses were used to determine potential predictors associated with the willingness to use placebo pills. Significance levels were set *α* = 0.05 in all analyses. Data were analyzed using IBM SPSS Statistics for Windows, Version 26.0 (Armonk, NY: IBM Corp). This study was approved by the Medical Ethics Committee of the St Antonius Hospital. The Medical Ethics Committee of the Amsterdam UMC agreed on participation. Participants provided informed consent for this study by filling out the questionnaires.

Four hundred seventy-eight questionnaires were sent to teenagers and 1166 to parents of patients, of which 751 (45%) questionnaires were completed: 226 (47%) teenagers and 525 (45%) parents. 656/751 (87%) respondents had also filled out the questions of the ancillary placebo study: 200/226 (88%) teenagers and 456/525 (87%) parents. Respondents’ departments included gastroenterology and hepatology (39%), psychosomatic disorder (10%), pulmonology (9%), endocrinology (7%), neurology (5%), urology (4%), infectious diseases (3%), cardiology (2%), and others (21%). Mean (SD) age of teenagers was 14.7 (1.8) years and 114 (57%) of the teenagers were girls. Mean (SD) age of patients for-which parents reported for was 9.7 (5.5) years; 87 (19%) of patients were aged < 4 years, 152 (33%) between 4 and 11 years, and 215 (47%) > 12 years. In total, 233 (51%) of these patients were girls.

**Fig. 1:**
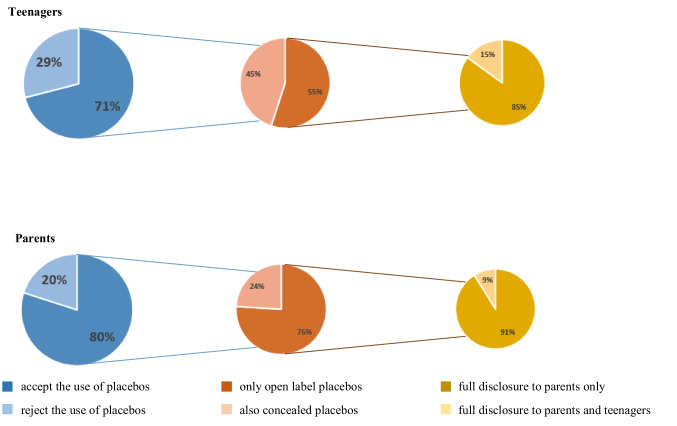
Preferences of respondents

Many participants (508 (77%)) were positive towards prescribing placebo medication: 143 (71%) teenagers vs. 365 (80%) parents (*χ*^2^(2) = 5.809, *p* =.019, Fig. 1). Significant more teenagers agreed on prescribing a placebo with deception (i.e., not disclosing that a placebo is being prescribed) compared to parents (64/143 (45%) vs. 89/365 (24%)), whereas 355/508 (70%) respondents reported that they were willing to receive placebo treatment but only when the physician would disclose that the treatment was a placebo (79/143 (55%) teenagers vs. 276/365 (76%) parents, *χ*^2^(1) = 20.259, *p* <.001). Majority of teenagers and parents agreed to disclose placebo treatment to parents exclusively; 252/276 (91%) of parents agreed to withhold information on the use of a placebo for the child vs. 67/79 (85%) of teenagers. Within the group of teenagers, increasing age was positively associated with the willingness for placebo pills (OR 0.803, 95%CI 0.659–0.979). A positive association was found with a higher level of parental education (OR 0.706, 95%CI 0.526–0.949). In both groups of respondents, sex, medication use, and a history of experienced side effects were not associated with the willingness to use placebo pills.

In the current study, we demonstrated that the majority (77%) of respondents – teenagers and parents – found it appropriate for healthcare professionals to prescribe placebo medication. Most teenagers and parents preferred that the physician fully discloses that the treatment is a placebo. Previous studies however have demonstrated lower treatment effects in patients receiving an open-label placebo than a concealed placebo [8, 11]. Compared to adult care, pediatrics has a unique feature that can solve this apparent paradox of pursuing the maximum effect using concealed placebos versus fulfilling patient’s wishes by being open about the treatment. The fact that parents are legally responsible for their children under the age of 12 years allows disclosing placebo treatment to the parents while concealing it for the young patient: a partly open-label placebo. The optimal advantages of placebo treatment can be achieved without legal infringements.

By law, the situation is different when treating teenagers. Here, clinicians cannot withhold information on placebo use, making an open-label placebo the only possible option for this patient group. Although the results are less compared to concealed placebo, several studies in both adults and children with IBS or functional abdominal pain have demonstrated that open-label placebo treatment can result in clinically meaningful symptom improvement compared to no treatment [12–14]. Moreover, some contradictions on treatment effects rates of open-label placebo versus concealed placebo interventions in the literature exist. Limited research suggests that open-label placebo interventions might not be inferior to concealed placebo treatment when provided with compelling rationale [15, 16]. Given the advantages of a placebo (i.e., reduced use of pharmacological therapies with less adverse effects), prescribing open-label placebos is an exciting strategy that needs further exploration in daily care for teenagers.

In the current study, we identified that increasing age and higher parental education were positively associated with the willingness for placebo pills. The possibility that adolescents and parents generally acquired more knowledge through the years about the concept of placebo before filling out our questionnaire may explain these results. In our study, the amount of information about the placebo effect was relatively scarce. Future studies are warranted to assess if more extensive education may positively influence parental and teenagers’ views on placebo usage.

A strength of this study is that we also explored teenagers’ attitudes towards prescribing placebos, while earlier studies investigated only parental attitudes. Secondly, this trial included teenagers and parents from both secondary and tertiary care centers in rural and urban areas, increasing the generalizability of the results. A limitation of this study is that we did not study the influence of ethnicity and culture. It could be hypothesized that differences exist in views on using placebo interventions between certain ethnic and cultural groups.

In conclusion, this study demonstrates the willingness of teenagers and parents of pediatric patients to receive placebo medication. Most respondents prefer open-label placebo. Especially for children under the age of 12 years, there is the possibility of disclosing placebo treatment to parents only. Placebo medication becoming more acceptable and integrated into daily care may contribute to a decrease in medication use.

## Data Availability

Deidentified individual participant data are available upon request.
